# Correction: Development and characterization of a babassu nut oil-based moisturizing cosmetic emulsion with a high sun protection factor

**DOI:** 10.1039/d0ra90080j

**Published:** 2020-07-23

**Authors:** Michael Jackson Ferreira da Silva, Alisson Mendes Rodrigues, Italo Rennan Sousa Vieira, Gelmires de Araújo Neves, Romualdo Rodrigues Menezes, Eloisa da Graça do Rosário Gonçalves, Maria Célia Pires Costa

**Affiliations:** Universidade Federal do Maranhão, Programa de Pós-Graduação em Saúde e Ambiente, Campus do Bacanga 65085-580 São Luís MA Brazil; Universidade Federal de Campina Grande, Centro de Ciências e Tecnologia, Unidade Acadêmica de Engenharia de Materiais 58109-970 Campina Grande PB Brazil; Universidade do Estado do Rio de Janeiro, Instituto de Química, Pavilhão Reitor Haroldo Lisboa da Cunha Maracanã 20550-900 Rio de Janeiro RJ Brazil; Universidade Federal do Maranhão, Departamento de Patologia, Centro de Ciências da Saúde, Campus do Bacanga 65085-580 São Luís MA Brazil; Universidade Estadual do Maranhão, Departamento de Química, Campus Universitário Paulo VI 65055-970 São Luís MA Brazil celiacosta@prof.elointernet.com.br

## Abstract

Correction for ‘Development and characterization of a babassu nut oil-based moisturizing cosmetic emulsion with a high sun protection factor’ by Michael Jackson Ferreira da Silva *et al.*, *RSC Adv.*, 2020, **10**, 26268–26276, DOI: 10.1039/D0RA00647E.

The authors regret that the name of one of the authors (Maria Célia Pires Costa) was shown incorrectly in the original article. The corrected author list is as shown above.

The Royal Society of Chemistry apologises for these errors and any consequent inconvenience to authors and readers.

## Supplementary Material

